# The burden of antimicrobial resistance in livestock: A framework to estimate its impact within the Global Burden of Animal Diseases programme

**DOI:** 10.1016/j.onehlt.2024.100917

**Published:** 2024-10-15

**Authors:** Sara Babo Martins, João Sucena Afonso, Christina Fastl, Benjamin Huntington, Jonathan Rushton

**Affiliations:** a Institute of Infection, Veterinary and Ecological Sciences, University of Liverpool, Liverpool, United Kingdom; b Global Burden of Animal Diseases Programme, Liverpool, United Kingdom; c Department of Epidemiology and Public Health, Sciensano, Brussels, Belgium; d Pengwern Animal Health Ltd, Merseyside, United Kingdom

**Keywords:** AMR, AMU, Livestock, One health, Social impact, Economic impact, Burden of disease, Conceptual framework

## Abstract

In addition to affecting animal health and production, antimicrobial resistance (AMR) in livestock can have far-reaching social and economic consequences, including on human health and the environment.

Given the diversity of data needs and the absence of standardised methodologies, the scale of antimicrobial use (AMU) and AMR's social and economic burden on livestock is complex to gauge. Yet, quantifying this impact can be an essential input for farm-level decision-making and, more widely, for policy development, public awareness, resource allocation to interventions and research and development prioritisation, particularly in a One Health context.

This work proposes a conceptual framework to guide the assessment of the burden of AMU and AMR in livestock using the Global Burden of Animal Diseases (GBADs) approach. Its development identified and mapped critical socio-economic concepts in AMU and AMR in livestock and their relationships. The Animal Health Loss Envelope (AHLE), a monetary metric that sets a boundary for overall losses from health hazards and allows an understanding of the relative importance of health problems in livestock, was used as the metric in which the concepts and data needs for the AMU and AMR assessment were anchored. The proposed framework identifies pathways for losses and data inputs needed to estimate the burden of AMU and AMR within this wider envelope of losses. These include information on health expenditure and mortality and morbidity effects related to AMR in livestock.

This work highlights the need for improved health and production data collection in livestock production as an essential stepping stone to accurately producing AMU and AMR burden estimates.

## Highlights

• The economic impact of AMU and AMR on livestock is unclear.
• It is also an unexplored aspect of AMR in a One Health context.
• Information on the burden of AMR in livestock burden can guide interventions.
• Critical data and parameters for these burden estimations can be identified through a conceptual framework.

## 1 Introduction

Antimicrobial resistance (AMR) is at the forefront of global health priorities, and initiatives to tackle this threat, including antimicrobial stewardship, infection prevention and biosecurity programmes, investments in research and development, and enhanced monitoring and surveillance, have been increasingly implemented [1]. As an archetype One Health issue, whose interconnections across humans, animals, plants and the wider environment are recognised as key, AMR mobilises stakeholders across all health sectors [2].

The increased urgency for action and allocation of resources to the AMR threat is partly driven by the growing understanding of AMR's social and economic burden. At the patient level, AMR prevents the efficient treatment of infections. Given the role of antimicrobials in modern medical care, for example, in routine medical procedures, AMR can cause far-reaching negative consequences [3]. Healthcare costs are also driven upwards due to therapeutic failures and treatment substitutions. From a broader economic perspective, productivity losses and societal impacts regarding equity, development and food security have also been described [[4], [5], [6]]. This understanding has been evolving with the steady expansion of empirical evidence on the economic impact of AMR on human health and healthcare systems. There is currently a better understanding of the cost of illness [7], the cost of inaction [8], and, recently, of the burden of morbidity and mortality expressed as Disability-Adjusted Life Years (DALYs) [9]. It has been estimated that 1.27 million deaths were due to bacterial AMR in 2019, placing AMR as a leading cause of death around the world, compared with other major global health priorities such as HIV and malaria [9]. Framing this evidence within a context of broader human health losses provides a powerful message, allowing the comparison of AMR's impact with other priority global health areas, informing trade-offs needed in resource allocation, and supporting complex decision-making [10,11].

In the animal health sector, however, evidence of AMR's burden and the ability to compare it with other animal health issues is currently missing. With a holistic One Health framing of AMR strategies underpinning action [12,13], broadening the perspective of the burden of AMR to include how AMR and AMU might also directly impact animals is critical. This information can improve our understanding of the costs and benefits of interventions in animal health and across other sectors from a One Health perspective.

This work contributes to filling this gap in knowledge regarding AMR's economic impact on livestock by proposing a conceptual framework to understand the burden of AMU and AMR in livestock. It examines how pathways for impact due to AMR and AMU in livestock can be framed within the Global Burden of Animal Diseases (GBADs) programme's analytical approach [14].

## 2 The building blocks: key concepts and context

### 2.1 AMU and AMR in livestock as expenditure and losses

Infectious diseases pose significant challenges to animal health, welfare and production. Economic consequences can be consequential, depending on the nature of the pathogen, production conditions, the mitigation strategies implemented and market circumstances (reviewed in [15,16]). Infectious diseases in livestock can have broader negative societal impacts, including on sustainable development, due to the socio-economic role animals have for farmers' livelihoods and in producing safe and affordable food. Zoonotic diseases in livestock also constitute a direct public health issue [17].

This importance is recognised at a societal level. It underpins public investments in animal health surveillance and disease control programmes [15], and at the farm level, it is the rationale for implementing and allocating resources to measures such as biosecurity [18,19]. Antimicrobials are an important part of the tool kit to mitigate the impact of infectious diseases at the farm level. They are used in livestock to prevent and treat infections and, in some countries, for growth promotion [20]. From an economic perspective, antimicrobials represent an input to animal production and a cost incurred to maximise production outputs by minimising infectious disease losses while enhancing animal and public health and welfare [21,22]. As a production input, antimicrobials have a specific characteristic important to its economic framing: they are drivers for AMR, meaning that the beneficial use of antimicrobials and their positive effects in infection mitigation may negatively affect their effectiveness in the long run [23]. These negative consequences tend not to be directly accrued on the user alone, creating instead losses to society as a whole [24]. AMR and the consequential loss of effectiveness of treatments have hence been framed as a negative externality of AMU [25,26].

The negative externalities on human health and the environment are critical contextual issues when considering AMR in livestock [27] as a key rationale for AMR mitigation investments in the animal health sector. From a One Health perspective, the assessment of AMR impact as a ‘super wicked’ problem [27] brings multiple challenges: from a complexly interlinked and insufficiently understood epidemiology across species and the environment to trade-offs with high stakes for health, food production, and sustainable development. These assessments require careful framing and a range of data inputs [[28], [29], [30], [31]]. As research with a One Health scope progresses [[32], [33], [34], [35]] and data generation and collection systems strengthen, the underpinning data for economic assessments with a more holistic outlook should become available and improve in quality.

### 2.2 GBADs analytical framework and the Animal Health Loss Envelope

The burden of AMU and AMR in livestock is part of a more comprehensive, multifactorial burden of diseases in livestock. Understanding this impact is at the core of the work developed by GBADs [14]. In the programme, the burden of animal disease is captured as losses due to disease and health problems and expenditure on preventative and reactive measures [36]. The programme's analytical framework encompasses a series of sequential steps that include understanding the biomass and economic value of livestock, estimating the AHLE at the farm level and its attribution to specific causes, and estimating the wider economic impact of animal disease [14].

The AHLE calculation is the GBAD's analytical framework step that quantifies what is spent and lost due to all health hazards. As a metric, it captures the difference in performance between the current situation and an ideal setting with no mortality, production losses or expenditure on health. [37]. For its calculation, a compartmental structure of the population of the study is modelled, considering biomass, fixed and variable production inputs, and animal production outputs, dependent on the animal species. The current setting models the production systems under present performance. The ideal setting is modelled by removing the effects of all health hazards and expenditures from the production system. The AHLE can then be quantified as the financial net change between the two scenarios – the current and the ideal setting [37]. As an overall envelope of losses, the resulting output overcomes a recurring problem in other animal health economics assessments: the risk of overestimating impact when single-cause diseases are assessed separately without accounting for co-morbidity and co-benefit effects [37,38]. These effects could be significant when assessing AMR.

The attribution process that follows allows an understanding of how each health hazard contributes to that envelope of burden [39]. This attribution can be done at different levels, from high-level causes to disease-specific causes. The three high-level attribution levels in the current GBADs attribution methods are infectious diseases, non-infectious causes and external issues. [41].

## 3 Conceptual framework: AMR and AMU in livestock within the GBADs analytical framework

While the GBADs framework provides overarching methods for all health threats, AMU and AMR pose specific challenges linked to how pathways for losses are generated. The conceptual framework presented in this manuscript helps clarify the connections between these various concepts and how these pathways are formed, guiding AMU and AMR-specific analysis in burden estimates following the GBADs analytical approach.

From the key concepts above, two aspects emerged that directly link AMR and AMU and the GBADs analytical steps:

i. AMU is a cost incurred to reduce infection-associated losses in livestock; hence, an animal health expenditure, part of the AHLE, and a component of the burden due to infectious diseases.

Other expenditures attributable to infectious diseases are linked to prevention, mitigation, or reduction of infection losses, such as improved biosecurity and vaccination. And,

ii. AMU may generate a negative externality—AMR, that produces mortality and morbidity directly attributable to resistant infections and, in turn, contributes to health expenditures in livestock.

**Fig. 1:**
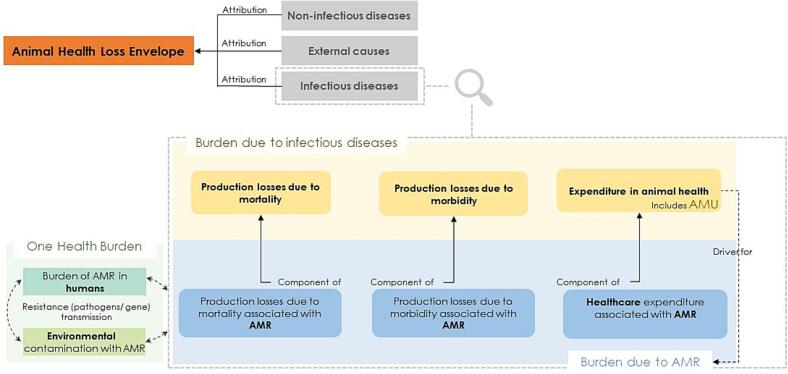
Conceptual framework depicting the concepts and their interlinkages for AMU and AMR burden within the AHLE and attribution steps of GBADs. From an attribution standpoint, the burden attributed to AMR contributes specifically to the infectious disease burden. While not part of the AHLE and attribution steps, the One Health perspective is critical when contextualising AMU and AMR burden in livestock.

When AMR is present, specific contributions to the broader infectious diseases component of losses are made in three ways. The first two ways are the mortality and morbidity effects of increased severity and duration of infectious illness in animals resulting from resistant pathogens. Those potentially adverse effects, ranging from potentially life-threatening to subclinical, are related to the inability to treat infections and have been described more extensively for animals kept for social reasons, sports, or breeding (reviewed in [40]). In contrast, evidence of the magnitude of these effects on livestock remains scarce. One estimate forecasts losses of up to 11 % on livestock productivity and exports of animal products due to restrictions imposed by trading standards due to AMR [5]. The third way is via expenditure incurred with treatment, treatment failures and worse or longer clinical outcomes. Healthcare expenditures associated with AMR include repeated treatments and treatments with potentially costlier therapeutic alternatives, additional diagnostic tests and animal health professionals' time. Furthermore, interventions implemented to mitigate the impact of AMR, such as outbreak control and costs with insurance [28,41], as well as stewardship initiatives, research investment, and monitoring and surveillance expenditures, can also be accrued in this component of the burden of AMR in livestock. Current knowledge quantifying these effects in livestock is also minimal. Fig. 1 provides a visual summary of the conceptual framework.

## 4 Towards a practical assessment: Identifying data needs in the assessment of the burden of AMU and AMR in livestock

The conceptual framework helps to identify the broad data categories needed to quantify the burden of AMR and AMU in livestock within the AHLE and attribution steps. To determine the exact data needs, the starting point of the assessment should carefully consider the scope. For livestock, various combinations between microorganisms and resistance against specific antimicrobials can be of interest, and practical considerations such as data availability or zoonotic potential are relevant in the decision. Identifying production systems and livestock species of interest should also be part of the scoping step since the AHLE data inputs and the clinical and production consequences of resistant infections are specific to species and production systems.

Following the GBADs analytical framework [14], and once the scope is established, estimating biomass and the AHLE are the initial steps. The details on the analytical steps and data inputs for biomass and the AHLE estimates are described elsewhere [[42], [43], [44]]. At this stage, data parameters on the valuation of the livestock enterprise, including prices and quantities of inputs and outputs, are already captured. This includes the data to estimate the expenditure with overall AMU.

Previous work [45] also describes the analytical steps in the attribution process in detail. To consider AMR specifically, data needs will extend from epidemiological parameters on AMR and its effects on health and production to specific expenditure data. Table 1 further details data needs per category. Importantly, while the public health costs of AMR are recognised as a critical externality, the framework presented focuses only on quantifying the burden on livestock.

**Table 1:** Data requirements for the assessment of the burden of AMU and AMR in livestock. The exact data inputs will depend on the scope of the assessment. Overall data requirements already collected for the AHLE estimate, a precursor step in this assessment, include, population, liveweight, reproduction parameters, mortality, outputs, fixed and variable inputs, market prices for outputs and inputs, data on the effects of disease on animals and production (e.g. stillborn animals, carcasses condemn at slaughter) and data for productivity changes under the ideal health scenario (e.g. carcass or milk yield). The assessment captures the burden of AMU and AMR and does not cover the attribution of AMR to its drivers, notably to AMU. *AMU for all causes, captured in the AHLE estimate; **includes AMU associated with resistant cases.

		Data needs	Possible data gaps
AMU*		Expenditure in antimicrobials according to the scope considered: - Antimicrobial use data - Retail price of antimicrobials	Antimicrobial use data, including at the granularity level required. Pricing data for antimicrobials.
AMR	Frequency measures	Incidence or prevalence data of AMR according to the scope considered.	Incidence or prevalence data at the granularity required.
	Production losses due to mortality	Mortality rate and disposal costs.	Mortality data attributed to drug-resistant infections.
	Production losses due to morbidity	Details will depend on the species and pathogen characteristics. Possible effects of AMR in production to capture can include losses in revenue due to: - Decreased feed conversion - Reduced growth - Delayed selling or withdrawal of products - Premature culling and replacement costs - Yield reduction - Delayed maturity and fertility	Frequency and impact data on the clinical and production effects of AMR
	Health expenditure	All expenditures incurred in the treatment and prevention of resistant cases**, including additional therapies, second-line treatments, diagnosis, and any AMR prevention and mitigation expenditures.	Clinical details associating treatments details with resistance. Pricing data for treatments and preventative measures. Information on diagnostics costs and purpose.

Table 1. Data requirements for attributing the burden of AMU and AMR in livestock. The exact data inputs will depend on the scope of the assessment.

Textbox 1 illustrates the data requirements for a set scope of assessment by using the example of the burden of mastitis caused by resistant pathogens in dairy cattle.

Textbox 1. Mastitis, an inflammation of the udder, negatively impacts health, welfare, and production in dairy animals, leading to significant economic losses [46,47]. Because mastitis is commonly caused by bacterial agents, antimicrobials are used for prevention and treatment. However, while mitigating the negative effects of mastitis, antimicrobials are a driver for the development of AMR, which ultimately can exacerbate losses due to treatment failures and worse clinical outcomes.

For this assessment, market prices for inputs (e.g., feed, labour) and outputs (e.g., milk price) are already captured in the calculation of the overall losses (AHLE). The calculation of the AHLE also captures expenditures associated with overall AMU to treat and prevent all infectious diseases in dairy. To attribute the burden of mastitis caused by resistant pathogens (RPs) in dairy cattle, data is needed on the prevalence or incidence of mastitis caused by RPs, its mortality and morbidity effects, and details on health expenditure undertaken to prevent or mitigate its effects. To establish the impact of resistance on mortality, specific data requirements would be frequency measures for mortality (e.g. due to toxaemia) and any animal replacement costs and disposal costs of the carcass specifically associated with mastitis caused by RPs. For morbidity effects, specific data requirements include any impact effects and/or length of impact in terms of quantity and quality of marketable milk, culling of the animal (e.g. due to systemic illness or udder damage), animal replacement costs, and potential reproduction effects (e.g. decreased conception, longer intervals between calving). Health expenditure is a product of the quantity of antimicrobials used to treat and prevent mastitis caused by RPs and its market price, including additional treatments, possibly with second-line molecules, to which adds any other treatment costs, diagnostics (e.g. to establish the reason for treatment failures), labour and extra prevention and mitigation activities (e.g. surveillance).

The assessment will yield two key outcomes: (1) the AHLE estimate, showing overall losses in dairy production due to all diseases through their effects on production; (2) the part of that envelope due specifically to mastitis caused by RPs. This can be expressed in absolute terms (i.e. a monetary cost) or relative terms (i.e. a proportion of the overall losses). The result will quantify the importance of mastitis caused by RPs in terms of overall losses in dairy and provide the baseline impact against which the benefits of investments can be assessed.

Alt-text: Unlabelled Box

## 5 Discussion

Our work proposes a conceptual framework for understanding the AMR and AMU-associated burden in livestock in the context of the GBADs approach. The quantification of the impact of AMU and AMR in livestock remains poorly understood and is an aspect of the One Health framing of AMR that is inadequately covered. This work aims to contribute to filling that knowledge gap with a conceptual tool to guide the assessment and identify data needs and potential data gaps for its practical use. While the framework is flexible enough to be applied broadly in a range of contexts and production systems, its parametrisation is context-specific and driven by the scope of the assessment.

By framing AMR and AMU-associated losses into the AHLE and attribution steps of GBADs, the framework seeks to place results within overall animal health losses. Unlike a single-cause disease study, it provides context for interpreting the results and, if reproduced across various contexts, a standardised process that yields comparable information [36]. Furthermore, while the AHLE and attribution steps of the GBADs analytical work remain focused on farm-level effects, extending the work in future iterations to AMR and AMU effects at the broader economy level following the analytical framework is possible. Inputs from the AHLE can be used for general and partial equilibrium models to evaluate the wider economic impacts [48].

We acknowledge that the range of data gaps expected for the framework's practical application is, currently, a significant challenge. Incomplete or inaccessible data is a common thread across work in AMR economics and potentially more so in AMR in animal health [30]. Production data, for example, can be deemed proprietary information and, hence, not be readily accessible to stakeholders outside of the industry. Diagnostic data concerning infectious diseases and AMR and their clinical and production consequences is also sparse. Consequently, data with a clinical or epidemiological link associating mortality and morbidity effects to antimicrobial-resistant pathogens is just about non-existent. The required granularity for AMU data, timeline of treatments, and pricing data can also represent a challenge. Differences between data availability across settings, from low-middle-income to high-income countries, and different animal species are also likely, adding complexity to the production and comparison of estimates more globally.

Data gaps are not exclusive to the economics of AMR, and sounder data for evidence and strengthening systems to capture that data are pillars of strategies to mitigate AMR [49]. Conceptual frameworks and their subsequent practical application can help address such data challenges by identifying data needs, pinpointing data gaps, and, in doing so, contributing to the design of data-generation and capturing systems. For example, to populate socio-economic estimates for livestock with empirical data, information captured should be expanded from epidemiological resistance data and use data to, when possible, population-level data on clinical and production parameters linked to resistance and animal health expenditure data. While data-capturing and generation capacity strengthens, developing the analysis by addressing gaps in other ways and carefully framing the scope to increase its feasibility remains possible [50]. One example is leveraging ongoing initiatives and projects that collect AMR and AMU data in livestock through careful stakeholder mapping and engagement as an opportunity to address these gaps. Afonso et al. [48] used the conceptual framework to guide stakeholders and data mapping exercises in Tanzania as a roadmap to identify overall data sources. Primary data collection through farmers' surveys is another way forward, as seen in previous work [51]. Further work will apply the framework in different settings, allowing the evaluation of its practical validity and applicability beyond the theoretical concepts.

Through continued efforts to address AMR data challenges in the livestock sector, it should become possible to understand, with greater accuracy, how the burden of AMU and AMR is produced and attributed and use such information to promote more sustainable animal health practices. Furthermore, where background data on who owns, manages, and works in the production systems are available, there will be an opportunity to leverage farm-level information to understand the broader social aspects of the impact of AMR.

## 6 Conclusion

Our work introduces a conceptual framework for assessing the burden of AMU and AMR in livestock within the broader context of the GBADs. We acknowledge the challenges of evaluating AMR's socio-economic impacts, including data gaps, and stress the importance of collaborative efforts and data accessibility. Addressing these challenges and leveraging existing initiatives will be crucial in advancing our understanding of AMR's impacts. The future application of the framework in specific contexts will allow us to test the approach and inform further iterations inductively.

## Funding sources

This work was funded by The Government of the United Kingdom of Great Britain and Northern Ireland, acting through the Department of Health and Social Care (DHSC) and the World Organization for Animal Health (WOAH).

## CRediT authorship contribution statement

**Sara Babo Martins:** Writing – original draft, Visualization, Methodology, Funding acquisition, Conceptualization. **João Sucena Afonso:** Writing – review & editing, Visualization, Funding acquisition, Conceptualization. **Christina Fastl:** Writing – review & editing, Visualization, Conceptualization. **Benjamin Huntington:** Writing – review & editing, Funding acquisition, Conceptualization. **Jonathan Rushton:** Writing – review & editing, Funding acquisition, Conceptualization.

## Declaration of competing interest

None.

## Data Availability

No data was used for the research described in the article.
